# Absence of MeCP2 binding to non-methylated GT-rich sequences *in vivo*

**DOI:** 10.1093/nar/gkaa102

**Published:** 2020-02-17

**Authors:** John C Connelly, Justyna Cholewa-Waclaw, Shaun Webb, Verdiana Steccanella, Bartlomiej Waclaw, Adrian Bird

**Affiliations:** 1 The Wellcome Centre for Cell Biology, University of Edinburgh, EH9 3BF Edinburgh, UK; 2 School of Physics and Astronomy, University of Edinburgh, EH9 3FD Edinburgh, UK

## Abstract

MeCP2 is a nuclear protein that binds to sites of cytosine methylation in the genome. While most evidence confirms this epigenetic mark as the primary determinant of DNA binding, MeCP2 is also reported to have an affinity for non-methylated DNA sequences. Here we investigated the molecular basis and *in vivo* significance of its reported affinity for non-methylated GT-rich sequences. We confirmed this interaction with isolated domains of MeCP2 *in vitro* and defined a minimal target DNA sequence. Binding depends on pyrimidine 5′ methyl groups provided by thymine and requires adjacent guanines and a correctly orientated A/T-rich flanking sequence. Unexpectedly, full-length MeCP2 protein failed to bind GT-rich sequences *in vitro*. To test for MeCP2 binding to these motifs *in vivo*, we analysed human neuronal cells using ChIP-seq and ATAC-seq technologies. While both methods robustly detected DNA methylation-dependent binding of MeCP2 to mCG and mCAC, neither showed evidence of MeCP2 binding to GT-rich motifs. The data suggest that GT binding is an *in vitro* phenomenon without *in vivo* relevance. Our findings argue that MeCP2 does not read unadorned DNA sequence and therefore support the notion that its primary role is to interpret epigenetic modifications of DNA.

## INTRODUCTION

The DNA base cytosine can exist in a variety of modified forms of which 5-methylcytosine (mC) is the most abundant in vertebrates (1). Cytosine methylation is implicated in regulation of a variety of molecular processes, including transcription and chromosome organization (2). In most cell types cytosine modification occurs almost exclusively at the dinucleotide CG, but in brain the dinucleotide CA is also highly methylated particularly within the trinucleotide CAC (3,4). The 5-methylcytosine binding protein MeCP2 is also present at high levels in neuronal cells (5) where it interacts with both methyl-CG (mCG) and methyl-CAC (mCAC) (6–8). A primary function of MeCP2 is to recruit the NCoR1/2 corepressor complex to these methylated sites and thereby restrain neuronal transcription (6,8–10). Mutations compromising either DNA binding or corepressor recruitment cause the severe neurological disorder Rett syndrome, emphasizing the importance of this role (9,11). Discrete protein domains responsible for methyl-CpG/mCAC binding (the methyl-binding domain: MBD) and NCoR1/2 interaction domain (NID) have been defined by deletion analysis and X-ray crystallography of the protein–DNA and protein–protein complexes (9,12–14). Importantly, these two domains alone are sufficient to rescue survival of MeCP2-deficient mice (15).

Most studies confirm the pivotal importance of DNA methylation in determining the MeCP2 interaction with chromatin (5,7,8,16,17), but evidence that other features of DNA sequence can be recognized has been presented (see (18)). These findings question the notion that MeCP2 is predominantly a ‘reader’ of the epigenetic DNA methylation mark. If non-methylated sites were to be prominent among its targets, MeCP2 could be viewed less as a reader of the epigenome, which varies in different developmental cell lineages, and more of a conventional transcription factor that interprets the unchanging DNA sequence. Thus, interpretation of MeCP2 function is strongly affected by whether it is instructed by the epigenome alone or also by the genomic base sequence. To investigate the significance of DNA methylation-independent binding, we chose to re-visit the best-characterized example of a specific non-methylated DNA sequence that is targeted by MeCP2. Early *in vitro* experiments established that an N-terminal fragment of chicken MeCP2 bound with high affinity to several DNA sequences that typically contained a GT-rich sequence, often flanked by an A/T-run (19,20). Recently a structure of the MeCP2 MBD in complex with GTG(T)-containing DNA has been solved (21). Using human MeCP2 we defined GT-rich sequences that can interact with domains of MeCP2 and showed that binding depends on guanine and the pyrimidine methyl group provided by thymine. Unexpectedly, the full-length protein failed to exhibit detectable DNA methylation-independent binding *in vitro*, suggesting that this may be a property only of MeCP2 sub-fragments. We therefore tested MeCP2 binding to GT-rich motifs *in vivo*. Using independent assays based on chromatin immunoprecipitation-sequencing (ChIP-seq) and transposase accessible chromatin sequencing (ATAC-seq), we were unable to detect this mode of MeCP2 binding, even when MeCP2 was expressed at high levels. These results suggest that GT-rich binding is an *in vitro* phenomenon that is not relevant *in vivo*. They therefore strengthen the likelihood that symmetrically methylated mCG and asymmetrically methylated mCAC are the primary recognition modules for MeCP2 in living cells.

## MATERIALS AND METHODS

### Recombinant MeCP2 expression and purification

**Figure 1.**
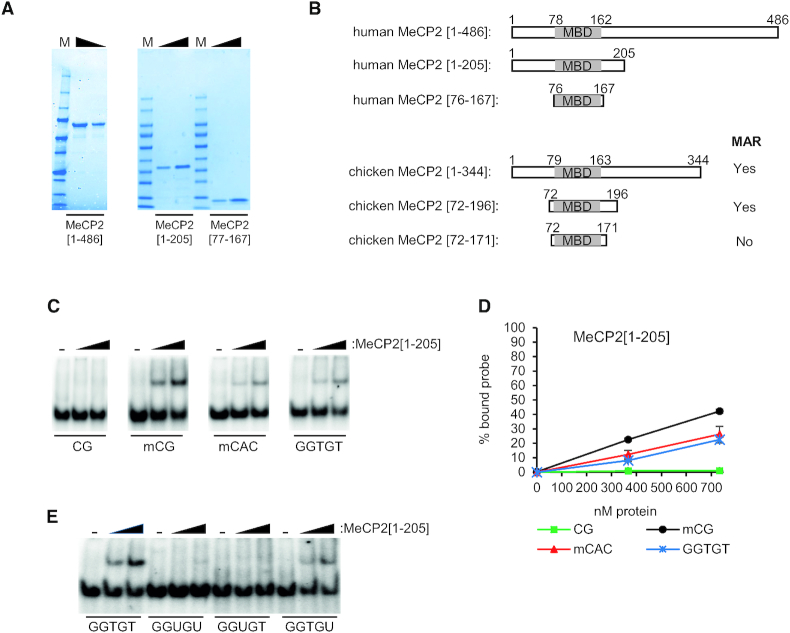
MeCP2 [1–205] binds to non-methylated GT-rich DNA *in vitro* and requires a methyl group provided by thymine. (**A**) Coomassie stained gel of purified recombinant proteins used in this study, full-length MeCP2[1–486], MeCP2[1–205] and MeCP2[77–167]. M = protein standard (Precision Plus, Bio-Rad). (**B**) Schematic diagram of the fragments of MeCP2 used here and in earlier studies. The lower diagrams show fragments of chicken MeCP2 tested previously for binding to GT-rich sequences (MAR) with high affinity, indicating whether GT binding was detected (20). The human MBD (grey shading) is located between amino-acids 78 and 162 (14). The highly homologous chicken MBD (also grey) extends from amino-acid 79 to 163. (**C**) EMSAs using varying amounts of MeCP2[1–205] or no protein (−) with probes containing non-methylated CG (CG), methylated CG (mCG), methylated CAC (mCAC) or GGTGT. (**D**) Graph showing quantification of MeCP2[1–205] binding to probes containing CG (filled squares), mCG (filled circles), mCAC (filled triangles) or GGTGT (crosses). Mean percentage of probe shifted (±SEM) based on triplicate experiments. (**E**) EMSAs using no protein (−) or varying amounts of MeCP2[1–205] with probes containing GGTGT, GGUGU, GGUGT or GGTGU to assess the influence of the thymine methyl group on binding.

Recombinant human MeCP2 protein was fused to a C-terminal histidine tag, to facilitate purification, and expressed from the vector pET30b. Plasmids expressing MeCP2[1-205]; MeCP2[77-167]; and MeCP2[1-486] were constructed as described previously (29). Proteins were produced in bacteria using standard procedures as described (22) (see Figure 1A).

### Oligonucleotide probes

Synthetic DNA oligonucleotides (Biomers, Germany) were based on a 58 bp parent probe derived from promoter III of the mouse *Bdnf* locus whose crystal structure in conjunction with MeCP2[77-167] has been solved (12). The sequence contains a central mCG motif followed at the 3′-end by an A/T-flank. In some experiments, the CG or A/T-flank were substituted with the sequences indicated in Table 1. Single-stranded oligonucleotides were annealed and end-labelled with T4 polynucleotide kinase (NEB) and ^32^P-γ-ATP (Perkin Elmer). For pull-down assays the parent DNA sequences were as described in (23) with the adjustments described in Table 1.

**Table 1.** Oligonucleotides used in this study

Name	Sequence of oligonucleotide
CG (parent)	5′-AAGCATGCAATGCCCTGGAA**CGG**AATTCTTCTAATAAAAGATGTATCATTTTAAATGC-3′
mCG	5′-AAGCATGCAATGCCCTGGAA**mCGG**AATTCTTCTAATAAAAGATGTATCATTTTAAATGC-3′
CAC	5′-AAGCATGCAATGCCCTGGAA**CAC**AATTCTTCTAATAAAAGATGTATCATTTTAAATGC-3′
mCAC	5′-AAGCATGCAATGCCCTGGAA**mCAC**AATTCTTCTAATAAAAGATGTATCATTTTAAATGC-3′
GGTGT	5′-AAGCATGCAATGCCCTGGAA**GGTGT**AATTCTTCTAATAAAAGATGTATCATTTTAAATGC-3′
GT_1_	5′-AAGCATGCAATGCCCTGGAA**GT**AATTCTTCTAATAAAAGATGTATCATTTTAAATGC-3′
GT_2_	5′-AAGCATGCAATGCCCTGGAA**GTGT**AATTCTTCTAATAAAAGATGTATCATTTTAAATGC-3′
GT_3_	5′-AAGCATGCAATGCCCTGGAA**GTGTGT**AATTCTTCTAATAAAAGATGTATCATTTTAAATGC-3′
GT_4_	5′-AAGCATGCAATGCCCTGGAA**GTGTGTGT**AATTCTTCTAATAAAAGATGTATCATTTTAAATGC-3′
GT_5_	5′-AAGCATGCAATGCCCTGGAA**GTGTGTGTGT**AATTCTTCTAATAAAAGATGTATCATTTTAAATGC-3′
ATGT	5′-AAGCATGCAATGCCCTGGAA**ATGT**AATTCTTCTAATAAAAGATGTATCATTTTAAATGC-3′
CTGT	5′-AAGCATGCAATGCCCTGGAA**CTGT**AATTCTTCTAATAAAAGATGTATCATTTTAAATGC-3′
TTGT	5′-AAGCATGCAATGCCCTGGAA**TTGT**AATTCTTCTAATAAAAGATGTATCATTTTAAATGC-3′
GTAT	5′-AAGCATGCAATGCCCTGGAA**GTAT**AATTCTTCTAATAAAAGATGTATCATTTTAAATGC-3′
GTCT	5′-AAGCATGCAATGCCCTGGAA**GTCT**AATTCTTCTAATAAAAGATGTATCATTTTAAATGC-3′
GTTT	5′-AAGCATGCAATGCCCTGGAA**GTTT**AATTCTTCTAATAAAAGATGTATCATTTTAAATGC-3′
GTGA	5′-AAGCATGCAATGCCCTGGAA**GTGA**AATTCTTCTAATAAAAGATGTATCATTTTAAATGC-3′
GTGC	5′-AAGCATGCAATGCCCTGGAA**GTGC**AATTCTTCTAATAAAAGATGTATCATTTTAAATGC-3′
GTGG	5′-AAGCATGCAATGCCCTGGAA**GTGG**AATTCTTCTAATAAAAGATGTATCATTTTAAATGC-3′
GGUGU	5′-AAGCATGCAATGCCCTGGAA**GGUGU**AATTCTTCTAATAAAAGATGTATCATTTTAAATGC-3′
GGUGT	5′-AAGCATGCAATGCCCTGGAA**GGUGT**AATTCTTCTAATAAAAGATGTATCATTTTAAATGC-3′
GGTGU	5′-AAGCATGCAATGCCCTGGAA**GGTGU**AATTCTTCTAATAAAAGATGTATCATTTTAAATGC-3′
GT_2_-inv bot	5′-AAGCATGCAATGCCCTGGAA**ACAC**AATTCTTCTAATAAAAGATGTATCATTTTAAATGC-3′
GT_2_-A/T mut	5′-AAGCATGCAATGCCCTGGAA**GTGT**AA**CG**CTTCT**CG**TA**CG**AGATGTATCATTTTAAATGC-3′
CG: pull-down template	5′-ACGTATATACGATTTACGTTATACGATTACGATATACGATTTACGTTAATACGTTTACGATTATTACGAATTTACGTTTTTACGAATATACGAAATACGTTTAATACGTAATTACGTATATTACGTATATACGATTTACGAATTACG-3′
CAC: pull-down template	5′-GCACATATGCACTTTGCACTATGCACTTGCACTATGCACTTTGCACTAATGCACTTGCACTTATTGCACATTTGCACTTTTGCACATATGCACAATGCACTTAATGCACAATTGCACATATTGCACATATGCACTTTGCACATTGCA-3′
GTGT: pull-down template	5′-GCACATATGCACTTTGCACTATGCACTTGTGTTATGTGTTTTGTGTTAATGTGTTTGTGTTTATTGTGTATTTGTGTTTTTGTGTATATGTGTAATGTGTTTAATGTGTAATTGTGTATATTGCACATATGCACTTTGCACATTGCA-3′
GGTGT: pull-down template	5′-GCACATATGCACTTTGCACTATGCACT**GGTGT**TA**GGTGT**TT**GGTGT**TAA**GGTGT**T**GGTG**TTTAT**GGTGT**ATT**GGTGT**TTT**GGTGT**ATA**GGTGT**AA**GGTGT**TTAA**GGTGT**AAT**GGTGT**ATATTGCACATATGCACTTTGCACATTGCA-3′

m = methylated. All molecules were annealed to the appropriate methylated or non-methylated reverse oligonucleotide.

### Electrophoretic mobility shift assay

Labelled DNA probe (1 ng) and 1 μg poly deoxyadenylic-thymidylic acid (polydA-dT) competitor (Sigma-Aldrich) were co-incubated on ice for 30 min with the indicated amount of MeCP2 in a 20 μl reaction volume containing 10 mM Tris–HCl, pH 7.5; 150 mM KCl, 0.1 mg/ml BSA; 5% glycerol; 0.1 mM EDTA. In the case of MeCP2[1–486], reactions were performed in 250 mM KCl (24). Samples were resolved on a chilled 10% TBE-acrylamide gel run at 100 V for 70 min in TBE. Gels were exposed to a phosphor screen overnight and imaged using a Typhoon FLA 9500 scanner (GE Healthcare). Where indicated the amount of probe bound by recombinant MeCP2 was quantified, in triplicate, using ImageJ software.

### DNA pull-down assay

This assay was essentially as described (23) with the following modifications. PCR-generated, biotin end-labelled 147 bp DNA probes (2 μg) were coupled to M280-streptavidin Dynabeads according to the manufacturer's instructions (Invitrogen). In the case of CG and CAC these motifs were either non-methylated or methylated (see Table 1 for sequences). Bead-DNA complexes were then co-incubated with 20 μg of rat brain nuclear protein extract (25) for 1.5 h at 4°C. Following extensive washing, bead-bound proteins were eluted using Laemmli buffer (Sigma) and resolved on a 4–15% SDS-polyacrylamide gel (NEB). The presence of MeCP2 was assayed by western blot using anti-MeCP2 monoclonal antibody M6818 (Sigma); with secondary detection employing IR-dye secondary antibodies (IRDye 800CW donkey anti-mouse, LI-COR Biosciences) then scanned using a LI-COR Odyssey machine.

### Generation of LUHMES cell lines expressing various levels of MeCP2

The procedure for culture and differentiation of the LUHMES (Lund Human Mesencephallic) cell line was previously described in (26). LUHMES cells expressing various levels of MeCP2 expression have been described in (10).

### Illumina sequencing and data analysis

The ChIP-seq, ATAC-seq and bisulfite mapping were performed as described (10). The data reported in this paper were deposited in the Gene Expression Omnibus (GEO) database, www.ncbi.nlm.nih.gov/geo (accession no. GSE125660). Trimmomatic version 0.32 was used to perform quality control on 94 and 75 bp paired-end reads to remove adapter sequence and unreliable reads for both TAB-seq and ChIP-seq. For TAB-seq, we used Bismark version 0.10 to further align and process the reads. Mapping was performed in bowtie2 mode to the human hg19 reference genome. Following alignment, duplicated reads were removed and methylation values were extracted as Bismark coverage and cytosine context files. We calculated the methylation percentage at each cytosine position as (mC/C)x100 and generated *.bed files for further processing. Bwa mem version 0.7.5 was used to map reads to the human hg19 reference genome. We filtered the alignments to remove reads that map to multiple locations in the genome and to blacklisted regions defined by the ENCODE project. We further removed duplicate reads with Picard version 1.107 MarkDuplicates (http://broadinstitute.github.io/picard/). To account for varying read depths we used deepTools version 2.5.1 to create bigWig files normalised by RPKM (reads per kilobase per million reads). To quantify MeCP2 occupancy on the genomic features of interest (mCG, mCA, GT, etc.), we rejected reads longer than 1 kb as alignment artefacts.

### ChIP-seq enrichment analysis

We used the EMBOSS tool dreg to find all instances of GT motifs in the hg19 reference sequence with a downstream run of at least two AT dinucleotides within thirteen bases. Overlapping motifs were merged and we selected 50 bases ± the start of each region using BEDTools. The average methylation state and read coverage of each region was calculated using bigWigAverageOverBed in conjunction with the processed BS-seq data. Regions with mean read coverage <10 were dropped and the remaining regions were subsetted based on the mean methylation percentage of all cytosine (mC%). Non-methylated regions were classified as mC% = 0 while methylated regions were defined as mC% > 10. We then plotted the relative enrichment of MeCP2 ChIP (WT and OE 11x) versus KO in 100 base bins across methylated and non-methylated regions for each GT motif as well as a set of control motifs (GGGTTT, TTTGGG). The relative enrichment is the log2 ratio of normalised read counts of MeCP2 ChIP versus KO ChIP scaled to the mean of the three flanking bins on either side of the plot. Next, we calculated the local GC% of each region by quantifying average GC% across the 2 kb plotted region. All filtering and plotting were performed in R using the base packages as well as genomation, seqplots and ggplot2.

### ATAC-seq footprint analysis

We obtained insertion profiles as described (10). The positions of insertions were accumulated to create insertion count profiles centred at different genomic features: (i) mCA, (ii) CA, (ii) GTGT, GGTGT (irrespectively of methylation) within 14 bases of a 3′AT-run as described in *Results*. To calculate the relative insertion probability profiles and remove Tn5 bias, we calculated

$$\begin{equation*}\ {f_i} = \ln \left[ {\frac{{{\rm{Nor}}{{\rm{m}}}\left( {n_i^{{\rm{sample}}}} \right)}}{{{\rm{Nor}}{{\rm{m}}}\left( {n_i^{{\rm{KO}}}} \right)}}} \right],\ \end{equation*}$$

where $n_i^{{\rm{sample}}}$ and $n_i^{{\rm{KO}}}$ are the insertion counts profiles for the MeCP2 OE 11x and MeCP2 KO, respectively, and ${\rm{Nor}}{{\rm{m}}_2}$ normalizes the counts profiles such that their flanks (–50…–41 bp and +41…50 bp) have values close to one:

$$\begin{equation*}{\rm{Nor}}{{\rm{m}}_2}\ \left( {{n_i}} \right) = \frac{{{n_i}}}{{\left( {\mathop \sum \nolimits_{j = - 50}^{ - 41} {n_j} + \mathop \sum \nolimits_{j = 41}^{50} {n_j}} \right)/20\ }}\ .\end{equation*}$$

To simulate ATAC-seq, we used the MeCP2 binding and ATAC-seq model from (10) (binding to motifs mCGx and mCAx where *x* = A, C, G, T, with different affinities) with the following changes: (i) we simulated the same number of insertions as in the experimental KO data, (ii) we simulated insertions in all chromosomes, (iii) we assumed that MeCP2 could also bind to non-methylated GTGT with a fraction $x$ of the mCG binding probability $p$. We simulated KO ($p\ = \ 0$) and OE 11x ($p\ = \ 0.063$ according to (10)).

To estimate the GTGT binding strength $x$ relative to mCG binding we used Bayesian inference. We previously showed (10) that motif occupancy can be estimated from the difference $r$ between relative insertion probabilities ${f_i}$ in the flanking regions and in the central region around the presumed binding site:

$$\begin{equation*}r\ = \frac{1}{{21}}\ \left( {\mathop \sum \limits_{i\ = \ - 10}^{10} {f_i}} \right) - \ \frac{1}{{142}}\left( {\mathop \sum \limits_{i\ = \ - 100}^{ - 30} {f_i} + \mathop \sum \limits_{i\ = \ 30}^{100} {f_i}} \right)\end{equation*}$$

We calculated $r$ for our data (mean of two replicates for OE 11x/KO) and used computer simulations described above to obtain Bayesian posterior probability distribution of $x$ that would give the same $r$, assuming uniform prior on $x$. In this analysis, we used all GGTGT motifs irrespectively of their methylation. We did this to increase the number of analysed motifs, as the number of GGTGT motifs devoid of methylation is only 20% of the total which would reduce the statistical power of our analysis. In fact, most motifs are only weakly methylated: 44% of all regions have mean methylation <10%, with less than 1% motifs having mean methylation >20%. Since our computer model explicitly includes MeCP2 binding to methylated C, any (small) contribution from mC binding to $r$ is accounted for when comparing the model and the experimental data, and does not bias the analysis.

## RESULTS

### Nucleotide determinants of cytosine methylation-independent DNA binding by MeCP2

To investigate the molecular basis of MeCP2 binding to non-methylated DNA *in vitro* we performed electrophoretic mobility shift assays (EMSAs) using a recombinant N-terminal fragment of MeCP2 comprising amino-acids 1–205 (see Figure 1A for gel analysis of proteins used in this study). MeCP2[1–205] includes the entire MBD (14) and sequences corresponding to a region of chicken MeCP2 (amino-acids 72 to 196) shown by Strätling and colleagues to bind non-methylated GT-rich DNA sequences (19,20) (Figure 1B). In comparative EMSAs, a non-methylated duplex probe containing GGTGT (Table 1) bound to MeCP2[1–205] (Figure 1C). By this semi-quantitative assay, the binding affinity was comparable to that of a probe containing mCAC, but lower than the classical MeCP2 recognition sequence mCG (Figure 1D). Our results confirm that binding of chicken MBD to this non-methylated sequence *in vitro* is replicated with the human protein.

A previous study showed that the interaction of MeCP2 with mCAC, whose complement is GTG, depends on the cytosine methyl-group, but also on the methyl group provided by thymine. Binding was abolished by substitution of T, which is base-paired to the central adenine of CAC, by uracil (U), which lacks the 5′ methyl group (8). We asked whether thymine methyl groups in the GGTGT sequence were also necessary for binding. Synthetic probes in which both thymines (T) were substituted with U (oligo:GGUGU) showed strongly impaired binding (Figure 1E). Probes in which only the central thymine was substituted by U (oligo:GGUGT) showed a similar reduction, suggesting that this methyl group is essential for binding, whereas mutating the 3′ T to U (oligo:GGTGU) had only a marginal effect. Thus, the pyrimidine methyl group on the central thymine of the pentanucleotide motif is critical for cytosine methylation-independent binding by MeCP2.

**Figure 2.**
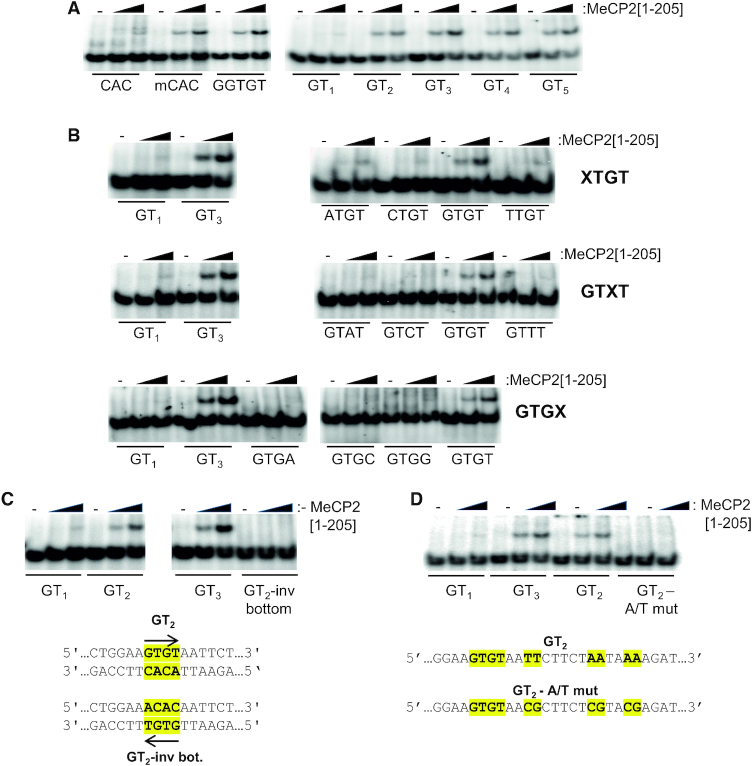
DNA sequence determinants of cytosine methylation-independent binding by MeCP2. (**A**) EMSAs using varying amounts of MeCP2[1–205] or no protein (−) with double-stranded oligonucleotide probes containing CAC, mCAC, GGTGT, GT_1_, GT_2_, GT_3_, GT_4_ or GT_5_ (see Table 1) to assess the influence of GT-length on binding. Note that CAC is the complement of GTG, indicating that this trinucleotide motif does not bind MeCP2[1–205]. (**B**) EMSAs in which MeCP2[1–205] was incubated with oligonucleotide probes altered at either the first (XTGT), third (GTXT), or fourth (GTGX) positions in the presence of varying amounts of MeCP2[1–205] or no protein (−). Probes containing GT_1_ or GT_3_ were used as negative and positive controls, respectively. (**C**) Determination of the effect of DNA sequences flanking GTGT binding to MeCP2. EMSAs using varying amounts of MeCP2[1–205] or no protein (−) with probes containing GT_1_, GT_2_, GT_3_ or GT_2_-inv bot (GT_2_-inverted indicated by yellow highlighting and inverted arrow). (**D**) The effect of altering the base composition of the 3′-AT-flank adjacent to GTGT was assayed by EMSAs using varying amounts of MeCP2[1–205] or no protein (−) with probes containing GT_1_, GT_2_, GT_3_ or GT_2_-A/T mut in which the TT-, AA- and AA-dinucleotides 3′-adjacent to GTGT were substituted by CG (yellow highlight).

As an N-terminal fragment of chicken MeCP2 can also bind to the sequence GTGTGT [GT_3_] (20), we used EMSAs to examine the effect of GT dinucleotide repeat length, from GT_1_ to GT_5_, on MeCP2[1–205] binding. Neither GT_1_ nor GTG (CAC on the complementary strand) bound significantly in EMSAs, but GT_2–5_ bound with similar affinity to GGTGT and mCAC, (Figure 2A). This suggests that GTGT is the minimal core MeCP2 target sequence *in vitro*. Alteration of bases at positions 1, 3 and 4 of this sequence showed that any deviation from GTGT greatly reduced binding by MeCP2[1–205] (Figure 2B). Background binding to CAC, base-paired with GTG, shows that the unmodified trinucleotide fails to interact by this assay (Figure 2A, B, bottom panels). In addition, we found that probe sequences flanking GTGT had a strong effect on *in vitro* binding affinity, as simply inverting the GTGT motif within an otherwise unchanged probe diminished binding (Figure 2C). Also, as reported for chicken MeCP2 (19), replacing a neighbouring AT-rich sequence in the original probe by a more GC-rich sequence greatly reduced the interaction (Figure 2D). The results demonstrate that the complex formed between MeCP2[1–205] and GT-rich DNA is highly sensitive to the flanking DNA sequences.

### Cytosine methylation-independent DNA binding requires specific fragments of MeCP2

**Figure 3.**
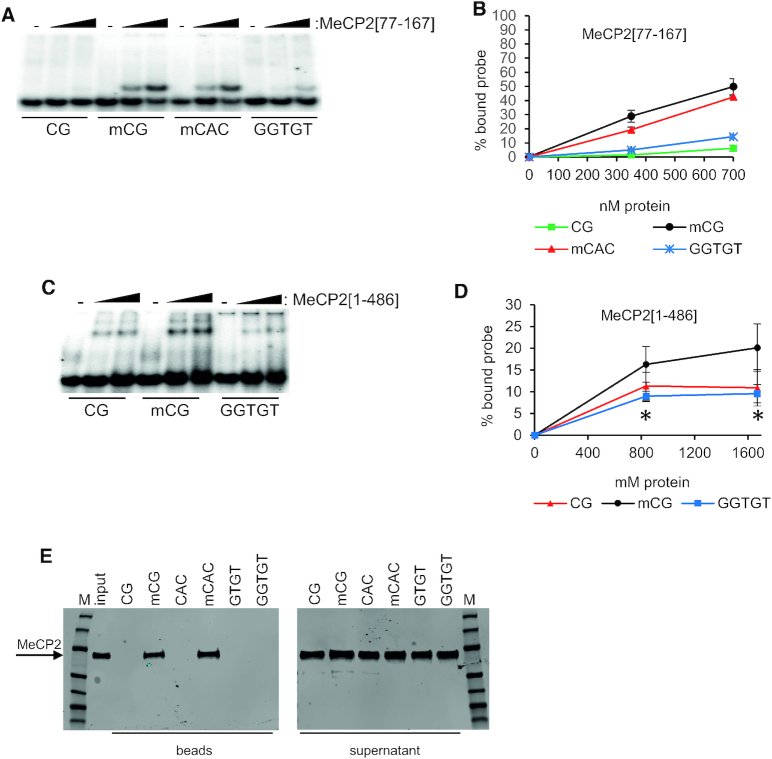
Cytosine methylation-independent binding requires specific fragments of MeCP2. (**A**) EMSA using varying amounts of MeCP2[77–167] or no protein (−) with probes containing non-methylated CG (CG), methylated mCG (mCG), methylated CAC (mCAC) or GGTGT. (**B**) Graph quantifying MeCP2[77–167] binding to probes containing CG (squares), mCG (circles), mCAC (triangles), or GGTGT (crosses). Mean percentage of probe shifted (±SEM) based on triplicate experiments. The results with the minimal MBD may be compared with those for MeCP2[1–205] in Figure 1C and D. (**C**) Full-length MeCP2[1–486] has a diminished ability to bind GGTGT. EMSAs using varying amounts of MeCP2[1–486] or no protein (−) with probes containing non-methylated CG, methylated CG (mCG), or GGTGT. (**D**) Graph showing quantification of MeCP2[1–486] binding to probes containing mCG (filled circles), non-methylated CG (filled triangles), or GGTGT (filled squares). Mean percentage of probe shifted (±SEM) based on triplicate experiments. Statistical significance was measured between mCG and GGTGT: *P < 0.05, **P < 0.01 and ***P < 0.001 (unpaired two-tailed t-test). The results with full-length MeCP2 may be compared with those for MeCP2[1–205] in Figure 1C and D. (**E**) Western blots with anti-MeCP2 antibody following DNA pull-down from rat brain nuclear extracts using immobilised DNA sequences containing CG, CAC, mCG, mCAC, GTGT, GGTGT. Left panel shows MeCP2 eluted from beads. Right panel shows that MeCP2 is present in all of the bead supernatants following pull-down. M = protein marker (Page-Ruler, Thermo Scientific).

GTGT binding, like mCG and mCAC binding, depends on a functional MBD, as mutation of the crucial arginine residue R111 to glycine abolished the interaction ((21) and data not shown). To determine more precisely whether the protein domains required for mCG binding and GGTGT binding are co-extensive we performed EMSAs using the minimal mCG-binding domain, MeCP2[77–167], whose structure in complex with methylated DNA was solved previously (12). MeCP2[77–167] bound to mCAC as expected, but the interaction with GGTGT was surprisingly reduced to near background levels (Figure 3A, B). These data agree with the earlier finding that that protein sequences immediately C-terminal to the minimal MBD are required for MeCP2 to interact with DNA in a mC-independent fashion *in vitro* (19). We next examined the ability of full-length MeCP2 to bind probes containing GGTGT, CG and mCG. As reported previously, MeCP2[1–486] shows reduced discrimination between mCG and CG in EMSAs compared with shorter MBD-containing fragments, as non-specific DNA binding increases using this assay (27). Despite this limitation, we detected a reproducible preference for binding to mCG compared with CG (Figure 3C, D). GGTGT-binding, however, was indistinguishable from that observed with non-methylated CG (Figure 3C, D). Due to the high background in the EMSA assay, we adopted an alternative ‘pull-down’ assay whereby brain extracts were incubated with PCR-generated probes containing multiple CG, CAC, mCG, mCAC, GTGT or GGTGT motifs (see Table 1 for sequences) that were immobilised on beads (23,25). Western blots detected strong retention of MeCP2 with mCG and mCAC, but no MeCP2 was recovered with CG, CAC, GTGT or GGTGT probes (Figure 3E). The results confirm that the affinity for GTGT seen with the MeCP2[1–205] sub-fragment *in vitro* is not a property shared by the intact protein.

### MeCP2 does not detectably bind to GT-rich sequences *in vivo*

The dependence of GT-motif binding on the surrounding DNA sequence context and on which domains of MeCP2 are tested made it critical to assess the relevance of this interaction *in vivo*. For this purpose, we interrogated MeCP2 ChIP-seq data derived from cultured human neurons (LUHMES cells) with varying levels of MeCP2 (10). These cells give rise to immature neurons with low levels of mCAC, which is advantageous when investigating MeCP2 binding to non-methylated CAC-containing motifs. A previous study showed enrichment of bound MeCP2 at mCG using ChIP-seq and also detected robust footprints at this methylated motif using ATAC-seq (10). We first searched the human reference genome for non-overlapping GTGT and GGTGT motifs with a 3′ run of A or T at least two base-pairs long within 13 bases and identified more than 10,000 examples of each (see Table 2). Excluding the few regions with low read coverage in whole genome bisulfite sequencing (TAB-seq) data (coverage < 10), motifs were then classified as either ‘non-methylated’ if no mC was detected within a 100 base-pair window surrounding the motif start position, or ‘methylated’ if >10% of mC was present. The analysis yielded several thousand motifs of each kind (Table 2).

**Table 2.** Number of GT-rich motifs analysed. Methylated and non-methylated columns show the number of sequences that passed the thresholds applied for read coverage, methylation level **etc** (see Materials and Methods). The Total column refers to the total number of GT-rich sequences found in the reference genome

	Methylated motif	Non-methylated motif	Total
*GGTGT*	7131	6049	153 588
*GTGT*	7130	5485	124 803

**Figure 4.**
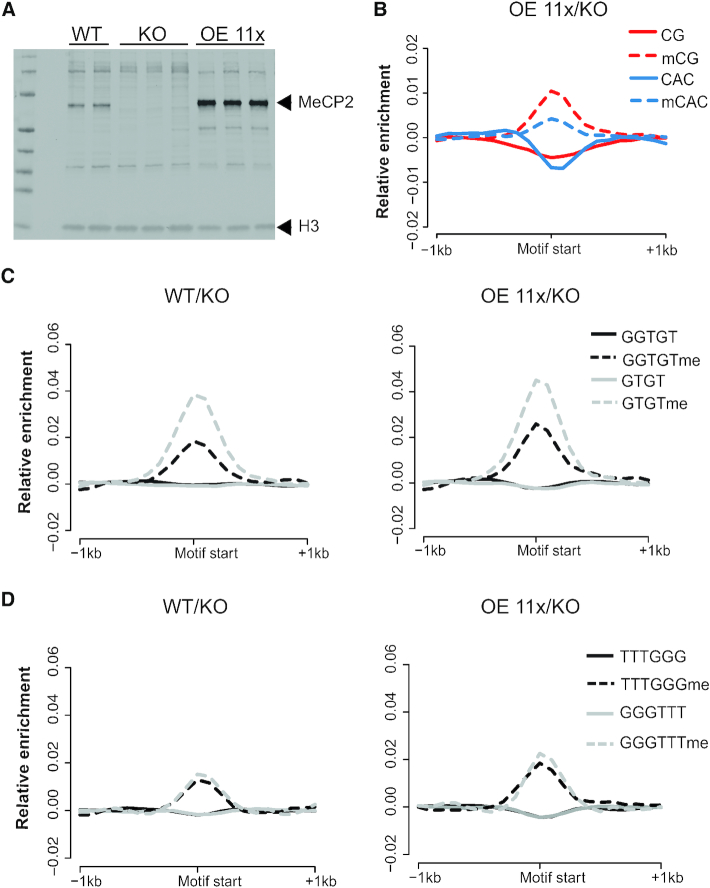
Native MeCP2 is not enriched at GT-rich sequences *in vivo*. (**A**) Western blot showing expression level of MeCP2 in WT, *MECP2* knockout (KO) and 11-fold over-expressing (OE 11x) human neuronal cells in culture (see reference (10)). Replicates are independent cloned cell lines. (**B**) Composite plots showing log_2_ enrichment of MeCP2 over windows containing methylated or unmethylated CG (red broken or solid lines, respectively) and methylated or unmethylated CAC (blue broken or solid lines, respectively) that are otherwise free of methylation. (**C**) Composite plots showing log_2_ enrichment of MeCP2 over GTGT- containing (grey) and GGTGT-containing (black) windows that do (dashed lines) or do not (solid lines) contain cytosine methylation elsewhere. WT (left panel) and OE 11x (right panel) reads are normalised to KO. (**D**) Composite plots showing log_2_ enrichment of MeCP2 over windows containing GGGTTT (grey) and TTTGGG (black) that do (dashed lines) or do not (solid lines) contain cytosine methylation elsewhere. WT (left panel) and OE 11x (right panel) are normalised to KO.

MeCP2-dependent ChIP enrichment at GT-rich motifs was tested by comparing MeCP2 ChIP-seq data from cells expressing wild-type levels of MeCP2, cells over-expressing MeCP2 at 11x the wild-type level, and cells in which the *MECP2* gene had been knocked-out (Figure 4A). A meta-analysis that plotted mean normalised ChIP-seq levels detected a peak of binding at both mCG and mCAC surrounded by a 100 base pair window that is otherwise non-methylated (Figure 4B). No peak of binding to non-methylated CG or CAC was present. A related analysis of GT-rich motifs lacking mC failed to detect MeCP2 binding in either WT or OE 11x cells. As a positive control, we found that GT-rich regions associated with one or more mCG or mCAC motifs within the surrounding 100 base pair window displayed a coincident MeCP2 ChIP peak (Figure 4C). A negative control was provided by the motifs GGGTTT and TTTGGG, which are not expected to bind MeCP2. They, like GT-rich motifs, failed to show ChIP peaks unless there was a methylated site nearby (Figure 4D). Summit analysis of ChIP peaks cannot be interpreted as a quantitative measure of binding, as we noted previously that peak height in ChIP-seq is not proportional to occupancy (10). Therefore, the variable peak heights associated with mCAC and GTGT when part of a methylated fragment do not imply differential affinities. Overall, the ChIP data offer no support for the notion that MeCP2 binds non-methylated GT-rich sequences *in vivo*.

**Figure 5.**
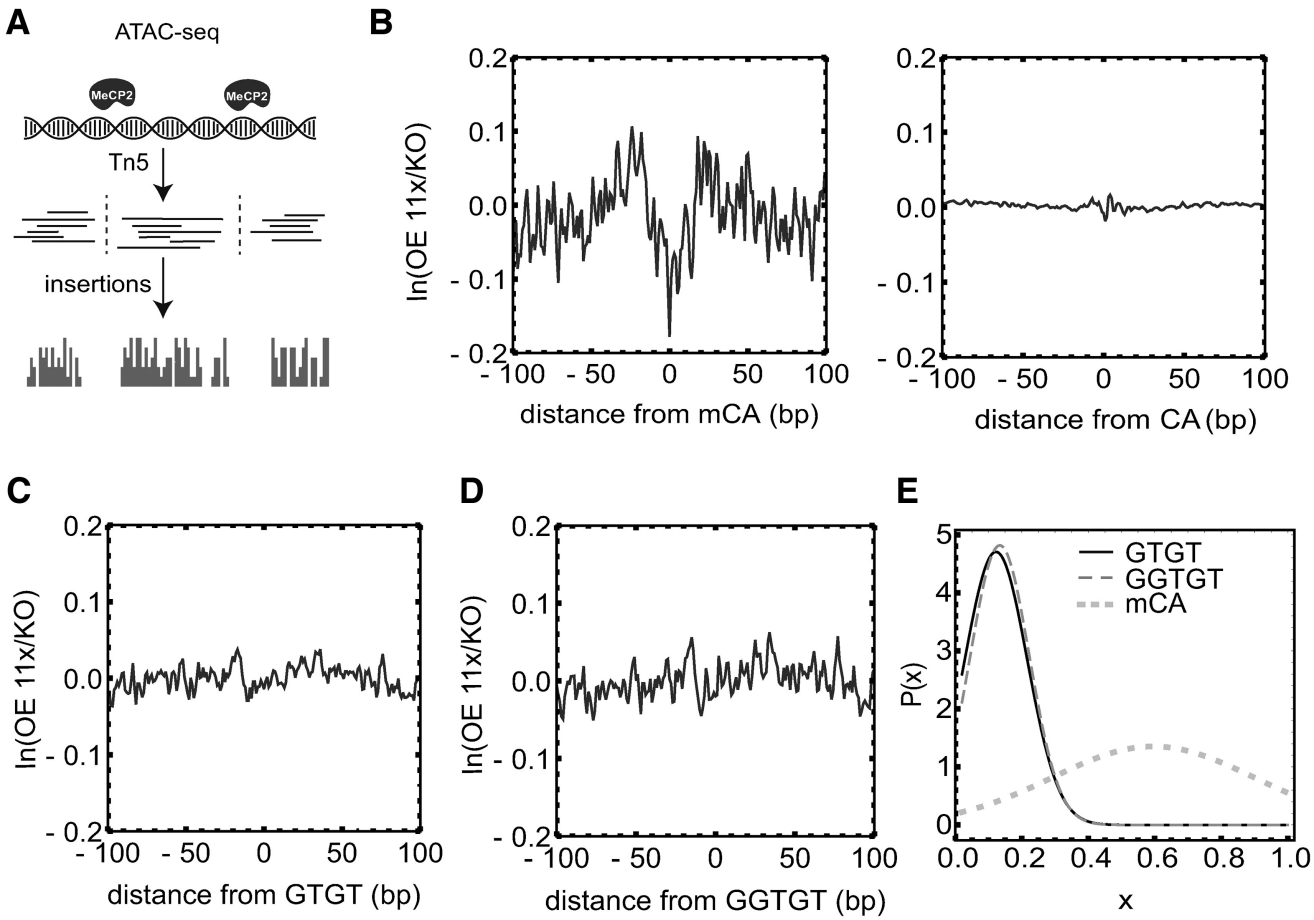
Absence of an ATAC-seq footprint of MeCP2 at GT-rich sequences in native chromatin. (**A**) A schematic representation of the ATAC-seq procedure and footprint analysis. (**B**) ATAC-seq insertion profile shows a MeCP2 footprint over mCA (left) but no footprint over non-methylated CA (right). (**C**) and (**D**) Absence of footprint at GTGT (left) and GGTGT (right), respectively. (**E**) Bayesian posterior probability distribution (P[x]) of the GTGT binding probability versus GTGT binding strength relative to mCG binding (x) that best reproduces the experimental ATAC-seq insertion profile. For comparison P[x] for mCA is shown (dotted line).

We complemented MeCP2 ChIP-seq enrichment analysis with an independent assay that relies on ATAC-seq *in vivo* footprint analysis (Figure 5A). Here a consistent position of bound MeCP2 is essential for visualisation of the footprint, as variably dispersed binding sites would not be detected. To validate the method, we first calculated enrichment profiles over methylated and non-methylated CA in LUHMES cells overexpressing MeCP2 11-fold. We previously showed a clear footprint over mCG in this cell line (10). As expected, the MeCP2 footprint is also observed over methylated CA (Figure 5B left), but absent at non-methylated CA (Figure 5B, right). If the *in vivo* MeCP2 binding to GTGT was as strong as the *in vitro* MBD binding, we would therefore expect to see a footprint on GTGT-containing sequences. To check this, we looked for MeCP2 footprints on all GTGT and GGTGT sequences, irrespective of methylation, and used computer modelling to factor out contributions from mCG and mCA binding. Figure 5C, D shows the absence of a footprint on GTGT and GGTGT in OE 11x LUHMES cells, in agreement with ChIP-seq *in vivo* data. Figure 5E shows the Bayesian posterior probability of MeCP2 binding GTGT with probability $x$ relative to that for mCG. The probability peaks close to zero which is consistent with absent or very weak binding to GTGT ($x < 0.29$ of that for mCG with 95% confidence).

## DISCUSSION

We re-investigated early reports that MeCP2 binds non-methylated GT-rich DNA sequences *in vitro* (19,20), which raised the possibility that GT-rich sequences are physiological ligands of MeCP2 (21). Our results confirmed that a sub-fragment of MeCP2 protein (MeCP2[1–205]) has a high affinity for the minimal target sequence GTGT. They also confirmed that binding depends upon a correctly orientated AT-rich flanking sequence and showed that a 5′ pyrimidine methyl group must be supplied by thymine. Unexpectedly we found that neither an isolated MBD polypeptide (MeCP2[77–167]) nor full-length MeCP2[1–486] supports the GT-rich mode of binding to a level above background *in vitro*. In apparent disagreement with this finding, a recent crystal structure of the minimal MBD complexed with a GTG(T)-containing DNA duplex has been determined (21). We note, however, that the dissociation constant reported for that interaction is 3–4 μM, which is an order of magnitude weaker than binding to mCG (21,28) and close to the background affinity of the MeCP2 MBD for any DNA sequence (28). Presumably this weak interaction was favoured by the high concentrations of DNA and protein in the crystallisation liquor. Our evidence that the minimal MBD shows background binding to GTGT is consistent with the published report (21) that GTGT binding by the isolated MBD *in vitro* is much weaker than its affinity for mCG or mCAC.

We found that MeCP2[1–205] showed GTGT binding, but MeCP2[77–167] (the minimal MBD) bound this sequence very weakly. Previous work showed that the addition of seven amino acids C-terminal to the minimal MBD reduced mC-dependent DNA binding and increased non-specific interactions with DNA (14). The recent structure of the low affinity (∼4 μM) complex between CAC (which is the complement of GTG) and the minimal MBD (21) does not explain why the extra amino acids C-terminal should facilitate binding of the 1–205 fragment or why this effect is lost in the full-length protein. Previously published structures, which only involve domains corresponding to the minimal MBD (77–167), suggest that DNA binding is accompanied by subtle conformational changes which may influence this interaction, but do not provide information on interactions with the rest of the protein (12,29). As our study revealed no evidence for GTGT binding to intact MeCP2, we do not consider that a detailed mechanistic understanding of this binding mode is a high priority.

Due to the inherent limitations of *in vitro* studies with purified components, we employed two independent *in vivo* assays to look for MeCP2 binding to GT-rich sequences *in vivo*. Using a human neuronal cell-line engineered to contain differing levels of MeCP2 we analysed ChIP-seq and ATAC-seq data (10). We verified that MeCP2 generates a ChIP peak and also a cytosine methylation-dependent footprint at the sequence mCAC, whose complement is GTG. Neither method of analysis detected evidence of MeCP2 binding to non-methylated GTGT or GGTGT motifs *in vivo*. In spite of this negative result, we cannot formally exclude the possibility that a subset of GT-rich sequences, below the detection limit of our assays, associates with MeCP2 *in vivo*. For example, changes to the sequence environment of GTGT may enhance binding. While we cannot disprove this possibility, several factors argue against it. Firstly, GGTGT is a subset of the simplest GTGT *in vitro* target sequence but tested negative for binding here. Secondly, for our *in vivo* analysis we imposed the condition that there must be a nearby AT-rich flanking sequence, as our evidence and the original work from the Strätling group (19) both indicated that a flanking AT-run aids *in vitro* binding by the MBD. Despite this attempt to enrich for favoured binding sites, we detected no MeCP2 footprints in native chromatin. Thirdly, we found that binding of full-length MeCP2 to this GT motif is indetectable using the sensitive pull-down assay for MeCP2 binding (Figure 3E). Thus, there is no *in vitro* precedent for an interaction of this kind involving the native protein *in vitro*. In the absence of experimental support for the notion that GT-rich sequences are physiological ligands of full-length MeCP2 *in vivo* or *in vitro*, the possibility that there is an undiscovered bound GT subset becomes highly speculative.

While these results affirm the importance of cytosine methylation for DNA binding by MeCP2, DNA sequence specificities other than GTGT have previously been considered. Recent reports suggested that GC-content rather than DNA methylation is the primary determinant of binding (30). As CpG islands are GC-rich but lack DNA methylation, this proposal conflicts with data from several laboratories showing that MeCP2 is depleted, not enriched, at these domains (6,8,10,25,31). It will be important to exclude the possibility that the intrinsic base compositional bias of DNA amplification and high throughput DNA sequencing contribute to this discrepancy. Two AT-hook motifs (32) contribute subtly to binding *in vivo* and *in vitro*, but appear to be dispensable, as polymorphisms that abolish the motifs occur in the population and mutations affecting them are absent in databases of clinically relevant mutations (33). In addition, to these known DNA binding domains, a non-specific affinity for DNA has been attributed to regions C-terminal to the NID (32). It is notable, however, that mice containing a radically truncated form of MeCP2 comprising only the MBD (a.a. 72–173) and NID (a.a. 272–312) are fully viable (15) suggesting that most of the protein, including the putative C-terminal DNA binding domain and AT-hooks, is non-essential.

As MeCP2 is a highly basic protein containing several disordered regions, it is important to distinguish non-specific DNA binding, for example due to electrostatic affinity to poly-anionic DNA, from those interactions that are specific and therefore more likely to be biologically relevant. This issue is illustrated by a chromatin immunoprecipitation study of mouse embryonic stem cells lacking DNA methylation, which found that MeCP2 binding redistributed to non-methylated sites in these cells (17). Mutations in the MBD that abolish or greatly reduce binding of MeCP2 to methylated DNA *in vitro* and *in vivo* nevertheless retained an association with chromatin. Despite persistence of chromatin binding, however, these mutant proteins cause Rett syndrome and are lethal in male mice. It follows that residual DNA methylation-independent binding cannot compensate for the absence of specific binding to methylated sites. Taken together, the data suggest that motifs containing 5-methylcytosine are the primary targets of MeCP2, predominantly in mCG and mCAC sites. Other modes of DNA binding, where confirmed, appear to be ancillary to this predominant DNA binding mode and consequently non-essential.

## DATA AVAILABILITY

The data reported in this paper were deposited in the Gene Expression Omnibus (GEO) database, www.ncbi.nlm.nih.gov/geo (accession no. GSE125660).

## References

[1] Hashimoto H., Zhang X., Vertino P.M., Cheng X. The mechanisms of generation, recognition, and erasure of DNA 5-Methylcytosine and thymine oxidations. J. Biol. Chem. 2015; 290:20723–20733. 26152719 10.1074/jbc.R115.656884 PMC4543634

[2] He Y., Ecker J.R. Non-CG methylation in the human genome. Annu. Rev. Genomics Hum. Genet. 2015; 16:55–77. 26077819 10.1146/annurev-genom-090413-025437 PMC4729449

[3] Lister R., Mukamel E.A., Nery J.R., Urich M., Puddifoot C.A., Johnson N.D., Lucero J., Huang Y., Dwork A.J., Schultz M.D. et al. Global epigenomic reconfiguration during mammalian brain development. Science. 2013; 341:1237905. 23828890 10.1126/science.1237905 PMC3785061

[4] Varley K.E., Gertz J., Bowling K.M., Parker S.L., Reddy T.E., Pauli-Behn F., Cross M.K., Williams B.A., Stamatoyannopoulos J.A., Crawford G.E. et al. Dynamic DNA methylation across diverse human cell lines and tissues. Genome Res. 2013; 23:555–567. 23325432 10.1101/gr.147942.112 PMC3589544

[5] Skene P.J., Illingworth R.S., Webb S., Kerr A.R., James K.D., Turner D.J., Andrews R., Bird A.P. Neuronal MeCP2 is expressed at near histone-octamer levels and globally alters the chromatin state. Mol. Cell. 2010; 37:457–468. 20188665 10.1016/j.molcel.2010.01.030 PMC4338610

[6] Kinde B., Wu D.Y., Greenberg M.E., Gabel H.W. DNA methylation in the gene body influences MeCP2-mediated gene repression. Proc. Natl. Acad. Sci. U.S.A. 2016; 113:15114–15119. 27965390 10.1073/pnas.1618737114 PMC5206576

[7] Gabel H.W., Kinde B., Stroud H., Gilbert C.S., Harmin D.A., Kastan N.R., Hemberg M., Ebert D.H., Greenberg M.E. Disruption of DNA-methylation-dependent long gene repression in Rett syndrome. Nature. 2015; 522:89–93. 25762136 10.1038/nature14319 PMC4480648

[8] Lagger S., Connelly J.C., Schweikert G., Webb S., Selfridge J., Ramsahoye B.H., Yu M., He C., Sanguinetti G., Sowers L.C. et al. MeCP2 recognizes cytosine methylated tri-nucleotide and di-nucleotide sequences to tune transcription in the mammalian brain. PLos Genet. 2017; 13:e1006793. 28498846 10.1371/journal.pgen.1006793 PMC5446194

[9] Lyst M.J., Ekiert R., Ebert D.H., Merusi C., Nowak J., Selfridge J., Guy J., Kastan N.R., Robinson N.D., de Lima Alves F. et al. Rett syndrome mutations abolish the interaction of MeCP2 with the NCoR/SMRT co-repressor. Nat. Neurosci. 2013; 16:898–902. 23770565 10.1038/nn.3434 PMC3786392

[10] Cholewa-Waclaw J., Shah R., Webb S., Chhatbar K., Ramsahoye B., Pusch O., Yu M., Greulich P., Waclaw B., Bird A.P. Quantitative modelling predicts the impact of DNA methylation on RNA polymerase II traffic. Proc. Natl. Acad. Sci. U.S.A. 2019; 116:14995–15000. 31289233 10.1073/pnas.1903549116 PMC6660794

[11] Amir R.E., Van den Veyver I.B., Wan M., Tran C.Q., Francke U., Zoghbi H.Y. Rett syndrome is caused by mutations in X-linked MECP2, encoding methyl-CpG-binding protein 2. Nat. Genet. 1999; 23:185–188. 10508514 10.1038/13810

[12] Ho K.L., McNae I.W., Schmiedeberg L., Klose R.J., Bird A.P., Walkinshaw M.D. MeCP2 binding to DNA depends upon hydration at methyl-CpG. Mol. Cell. 2008; 29:525–531. 18313390 10.1016/j.molcel.2007.12.028

[13] Kruusvee V., Lyst M.J., Taylor C., Tarnauskaite Z., Bird A.P., Cook A.G. Structure of the MeCP2-TBLR1 complex reveals a molecular basis for Rett syndrome and related disorders. Proc. Natl. Acad. Sci. U.S.A. 2017; 114:E3243–E3250. 28348241 10.1073/pnas.1700731114 PMC5402415

[14] Nan X., Meehan R.R., Bird A. Dissection of the methyl-CpG binding domain from the chromosomal protein MeCP2. Nucleic Acids Res. 1993; 21:4886–4892. 8177735 10.1093/nar/21.21.4886 PMC311401

[15] Tillotson R., Selfridge J., Koerner M.V., Gadalla K.K.E., Guy J., De Sousa D., Hector R.D., Cobb S.R., Bird A. Radically truncated MeCP2 rescues Rett syndrome-like neurological defects. Nature. 2017; 550:398–401. 29019980 10.1038/nature24058 PMC5884422

[16] Ishibashi T., Thambirajah A.A., Ausio J. MeCP2 preferentially binds to methylated linker DNA in the absence of the terminal tail of histone H3 and independently of histone acetylation. FEBS Lett. 2008; 582:1157–1162. 18339321 10.1016/j.febslet.2008.03.005

[17] Baubec T., Ivanek R., Lienert F., Schubeler D. Methylation-dependent and -independent genomic targeting principles of the MBD protein family. Cell. 2013; 153:480–492. 23582333 10.1016/j.cell.2013.03.011

[18] Lyst M.J., Bird A. Rett syndrome: a complex disorder with simple roots. Nat. Rev. Genet. 2015; 16:261–275. 25732612 10.1038/nrg3897

[19] Buhrmester H., von Kries J.P., Stratling W.H. Nuclear matrix protein ARBP recognizes a novel DNA sequence motif with high affinity. Biochemistry. 1995; 34:4108–4117. 7696275 10.1021/bi00012a029

[20] Weitzel J.M., Buhrmester H., Stratling W.H. Chicken MAR-binding protein ARBP is homologous to rat methyl-CpG-binding protein MeCP2. Mol. Cell Biol. 1997; 17:5656–5666. 9271441 10.1128/mcb.17.9.5656 PMC232414

[21] Lei M., Tempel W., Chen S., Liu K., Min J. Plasticity at the DNA recognition site of the MeCP2 mCG-binding domain. Biochim. Biophys. Acta Gene Regul. Mech. 2019; 1862:194409. 31356990 10.1016/j.bbagrm.2019.194409

[22] Brown K., Selfridge J., Lagger S., Connelly J., De Sousa D., Kerr A., Webb S., Guy J., Merusi C., Koerner M.V. et al. The molecular basis of variable phenotypic severity among common missense mutations causing Rett syndrome. Hum. Mol. Genet. 2016; 25:558–570. 26647311 10.1093/hmg/ddv496 PMC4731022

[23] Piccolo F.M., Liu Z., Dong P., Hsu C.L., Stoyanova E.I., Rao A., Tjian R., Heintz N. MeCP2 nuclear dynamics in live neurons results from low and high affinity chromatin interactions. Elife. 2019; 8:e51449. 31868585 10.7554/eLife.51449 PMC6957317

[24] Khrapunov S., Tao Y., Cheng H., Padlan C., Harris R., Galanopoulou A.S., Greally J.M., Girvin M.E., Brenowitz M. MeCP2 binding cooperativity inhibits DNA Modification-Specific recognition. Biochemistry. 2016; 55:4275–4285. 27420643 10.1021/acs.biochem.6b00451 PMC6532394

[25] Mellen M., Ayata P., Heintz N. 5-hydroxymethylcytosine accumulation in postmitotic neurons results in functional demethylation of expressed genes. Proc. Natl. Acad. Sci. U.S.A. 2017; 114:E7812–E7821. 28847947 10.1073/pnas.1708044114 PMC5604027

[26] Scholz D., Poltl D., Genewsky A., Weng M., Waldmann T., Schildknecht S., Leist M. Rapid, complete and large-scale generation of post-mitotic neurons from the human LUHMES cell line. J. Neurochem. 2011; 119:957–971. 21434924 10.1111/j.1471-4159.2011.07255.x

[27] Klose R.J., Sarraf S.A., Schmiedeberg L., McDermott S.M., Stancheva I., Bird A.P. DNA binding specificity of MeCP2 due to a requirement for A/T sequences adjacent to methyl-CpG. Mol. Cell. 2005; 19:667–678. 16137622 10.1016/j.molcel.2005.07.021

[28] Valinluck V., Tsai H.H., Rogstad D.K., Burdzy A., Bird A., Sowers L.C. Oxidative damage to methyl-CpG sequences inhibits the binding of the methyl-CpG binding domain (MBD) of methyl-CpG binding protein 2 (MeCP2). Nucleic Acids Res. 2004; 32:4100–4108. 15302911 10.1093/nar/gkh739 PMC514367

[29] Heitmann B., Maurer T., Weitzel J.M., Stratling W.H., Kalbitzer H.R., Brunner E. Solution structure of the matrix attachment region-binding domain of chicken MeCP2. Eur. J. Biochem. 2003; 270:3263–3270. 12869202 10.1046/j.1432-1033.2003.03714.x

[30] Rube H.T., Lee W., Hejna M., Chen H., Yasui D.H., Hess J.F., LaSalle J.M., Song J.S., Gong Q. Sequence features accurately predict genome-wide MeCP2 binding in vivo. Nat. Commun. 2016; 7:11025. 27008915 10.1038/ncomms11025 PMC4820824

[31] Chen L., Chen K., Lavery L.A., Baker S.A., Shaw C.A., Li W., Zoghbi H.Y. MeCP2 binds to non-CG methylated DNA as neurons mature, influencing transcription and the timing of onset for Rett syndrome. Proc. Natl. Acad. Sci. U.S.A. 2015; 112:5509–5514. 25870282 10.1073/pnas.1505909112 PMC4418849

[32] Baker S.A., Chen L., Wilkins A.D., Yu P., Lichtarge O., Zoghbi H.Y. An AT-hook domain in MeCP2 determines the clinical course of Rett syndrome and related disorders. Cell. 2013; 152:984–996. 23452848 10.1016/j.cell.2013.01.038 PMC3641682

[33] Lyst M.J., Connelly J., Merusi C., Bird A. Sequence specific DNA binding by AT-hook motifs in MeCP2. FEBS Lett. 2016; 590:2927–2933. 27461740 10.1002/1873-3468.12328 PMC5028900

